# A Mixture of D-Amino Acids Enhances the Biocidal Efficacy of CMIT/MIT Against Corrosive *Vibrio harveyi* Biofilm

**DOI:** 10.3389/fmicb.2020.557435

**Published:** 2020-09-04

**Authors:** Xiaomeng Liu, Zhong Li, Yongqiang Fan, Yassir Lekbach, Yongbo Song, Dake Xu, Zhichao Zhang, Lei Ding, Fuhui Wang

**Affiliations:** ^1^School of Life Sciences and Biopharmaceuticals, Shenyang Pharmaceutical University, Shenyang, China; ^2^College of Life and Health Sciences, Northeastern University, Shenyang, China; ^3^Shenyang National Laboratory for Materials Science, Northeastern University, Shenyang, China; ^4^Key Laboratory for Anisotropy and Texture of Materials (Ministry of Education), School of Materials Science and Engineering, Northeastern University, Shenyang, China; ^5^Shenyang Aircraft Design and Research Institute, Shenyang, China

**Keywords:** D-amino acid, D-tyrosine, D-methionine, *Vibrio harveyi*, biocide

## Abstract

Biocides are widely used for the mitigation of microbial contamination, especially in the field of the aviation fuel industry. However, the long-term use of biocide has raised the concerns regarding the environmental contamination and microbial drug resistance. In this study, the effect of a mixture of D-amino acids (D-tyrosine and D-methionine) on the enhancement of the bactericidal effect of 5-Chloro-2-Methyl-4-isothiazolin-3-one/2-Methyl-2H-isothiazole-3-one (CMIT/MIT) against corrosive *Vibrio harveyi* biofilm was evaluated. The results revealed that D-Tyr and D-Met alone can enhance the biocidal efficacy of CMIT/MIT, while the treatment of 5 ppm CMIT/MIT, 1 ppm D-Tyr and 100 ppm D-Met showed the best efficacy comparable to that of 25 ppm CMIT/MIT alone. The triple combination treatment successfully prevented the establishment of the corrosive *V. harveyi* biofilm and effectively removed the mature *V. harveyi* biofilm. These conclusions were confirmed by the results of sessile cell counts, images obtained by scanning electron microscope and confocal laser scanning microscope, and the ATP test kit.

## Introduction

The problem of microbial contamination of aircraft fuel tanks has been noticed by the international aviation industry for several decades (Skribachilin et al., 1993; Passman, 2013). There are many reports about microbial contamination of aircraft fuel tanks (Rajasekar and Ting, 2010; Passman, 2013). In 1958, the crash of an American Air Force bomber was due to the blockage of the fuel filter system by microbial biofilms (Rauch et al., 2006). International aviation accidents occur every year due to the microbial contamination of fuel system (Kosseva and Stanchev, 1994; McNamara et al., 2005; Cole and Paterson, 2009). To solve this issue, biocides have been widely used to control the microbial contamination in the field of aviation fuel systems (Bücker et al., 2014). Among the diversified biocides, 5-Chloro-2-Methyl-4-isothiazolin-3-one/2-Methyl-2H-isothiazole-3-one (CMIT/MIT) is commonly used in the fuel systems because of its broad spectrum and excellent compatibilities with surfactants and emulsifiers (Passman, 2013; Zimmer et al., 2013). However, many concerns regarding environmental contamination and microbial drug resistance caused by overuse and abuse of biocides have been raised (Russell, 2003). In view of this, the exploration of environmentally friendly methods for biofilm control and mitigation is desired.

In the natural environments, microbes prefer to aggregate together, forming biofilms rather than staying in planktonic forms. Sessile cells are encapsulated in the secreted extracellular polymeric substances (EPSs) which are mainly composed of extracellular nucleic acids, proteins and polysaccharides. The diffusion of antimicrobial agents were significantly prevented because of the biosurfacants and bioemulsifiers secreted into the EPSs (Billings et al., 2013; Abdallah et al., 2014). Moreover, the microbial metabolisms were also slowed down for the cells embedded in the biofilms, which decreased the absorption rates of antimicrobial agents (Wentland et al., 1996; Singh et al., 2017). Many stress resistance genes were also upregulated, accelerating the degradation and efflux pumping of antimicrobial agents. These reasons caused the dosage of antimicrobial agents used for mitigating biofilms 10–1000 fold higher than that used for killing planktonic bacteria (Gao et al., 2011; Sanchez et al., 2014). Consequently, the development of biocide enhancers that can improve the antimicrobial efficiency is of great importance in reducing usage and dosage of antimicrobial agents.

In recent years, D-amino acids have been noticed as biocide enhancers and played vital roles in the control and decomposition of microbial biofilms (Kolodkin-Gal et al., 2010; Sanchez et al., 2013; Ramon-Perez et al., 2014; Ampornaramveth et al., 2018). Since D-amino acids are widely distributed in nature and are degradable by many biological species, they are regarded as green and reliable biocide enhancers (Xu et al., 2012, 2014; Gittens et al., 2013; Li et al., 2016; Jia et al., 2017b). It has been reported that D-methionine can enhance the bactericidal efficacy of tetrakis hydroxymethyl phosphonium against *Desulfovibrio vulgaris* biofilm (Xu et al., 2014). Besides, D-tyrosine and D-Met have been proved to enhance the bactericidal efficacy of alkyldimethylbenzylammonium chloride against *D. vulgaris* biofilm (Jia et al., 2017a). Consequently, the expandation of D-amino acids as biocide enhancers in fuel systems for biofilm control and mitigation should possess a crucial application potential, which has not been reported yet.

In previous reports, it has been found that *Vibrio harveyi* may contaminate the aircraft fuel system (Ji-Dong et al., 1998; Passman, 2013), but there is no report about the corrosivity of *Vibrio harveyi* to the best of our knowledge. Herein, we firstly confirmed the corrosivity of *V. harveyi*, a common contamination microorganism, against the aluminum alloy 2024 (AA2024) which is a kind of typical materials used for fuel tank. Then, the effect of D-amino acids on the enhancement of the bactericidal effect of CMIT/MIT against *V. harveyi* biofilm was evaluated. The present work aimed to confirm the biocide enhancer role of the mixture of two D-amino acids, which can convert the difficult-to-kill biofilm into the planktonic counterparts, achieving removal of *V. harveyi* biofilm at a low dosage of CMIT/MIT.

## Materials and Methods

### Bacterium, Culture Medium, and Chemicals

The *V. harveyi* strain (CGMCC 1.1601) was purchased from China General Microbiological Culture Collection Center. 2216E culture medium was used to cultivate the bacterial strain, which was purchased from Qingdao Hope Bio-technology Co. (Qingdao, China). The main components of 2216E medium were (g/L): 1.8 CaCl_2_, 19.45 NaCl, 3.24 Na_2_SO_4_, 0.16 Na_2_CO_3_, 0.034 SrCl_2_, 0.08 KBr, 5.98 MgCl_2_, 0.08 SrBr_2_, 0.022 H_3_BO_3_, 0.0024 NaF, 0.004 Na_2_SiO_3_, 0.55 KCl, 0.0016 NH_4_NO_3_, 0.008 NaH_2_PO_4_, 5.0 peptone, 1.0 yeast extract and 0.1 ferric citrate. The bacterium was incubated at 28°C for cultivation, immersion test, and electrochemical test. *V. harveyi* was cultured in 2216E medium at 28°C for 12 h. The 2216E medium was used to simulate the worst-case scenario of microbial contamination.

D-Tyr, D-Met, and CMIT/MIT were purchased from Sigma-Aldrich (St. Louis, MO, United States). D-amino acids were filter-sterilized with a 0.22 μm membrane before use. The aluminum alloy 2024 (AA2024) coupons, with an exposed surface area of 1.0 cm × 1.0 cm, were abraded using different grades of silicon carbide papers (240, 400, and 600 grits), cleaned with absolute ethanol, air-dried, and sterilized under UV lights for 20 min before being immersed in the culture medium. The experiment test matrix is listed as follow: (A) no treatment (control), (B) 1 ppm D-Tyr, (C) 100 ppm D-Met, (D) 5 ppm CMIT/MIT, (E) 5 ppm CMIT/MIT + 1 ppm D-Tyr, (F) 5 ppm CMIT/MIT + 100 ppm D-Met, (G) 5 ppm CMIT/MIT + 1 ppm D-Tyr + 100 ppm D-Met, and (H) 25 ppm CMIT/MIT. All the experiments were performed in triplicate to guarantee the reproducibility.

### Electrochemical Analyses

The three-electrode system connected with a electrochemical workstation (Reference 600, Gamry Instruments, Inc., United States) was used to perform the electrochemical measurements. The three-electrode system contained a platinum sheet as the counter electrode, a saturated calomel electrode (SCE) as the reference electrode, an AA2024 coupon as the working electrode, and a 500 mL glass cell filled with 200 mL 2216E medium. *V. harveyi* at a concentration of approximately 10^6^ cells/mL was inoculated. The electrochemical cells were incubated at 28°C in an water bath. Linear polarization resistance (LPR) was measured by scanning −5 mV to +5 mV vs. *E*_*OCP*_ at 0.33 mV s^–1^ every day, and the potentiodynamic polarization was performed at the end of immersion for 7 days.

### Biofilm Prevention Test

Three AA2024 coupons were put in 200 mL conical flasks containing 100 mL culture media. Different treatment chemicals at different concentrations were added into the culture medium. The inoculated concentration of *V. harveyi* was approximately 10^6^ cells/mL. The flasks were incubated at 28°C for 3 days before the coupons were taken out for cell enumeration analysis.

### Biofilm Removal Test

The AA2024 coupons were incubated with *V. harveyi* in 200 mL conical flasks containing 100 mL culture media for 3 days to form mature biofilms. After that, the coupons were taken out and rinsed in 0.9% NaCl solution to remove the loose planktonic cells. Then, they were transferred into a 24-well plate containing 2 mL of culture medium with different treatment chemicals, and further incubated at 28°C for 24 h. After that, the coupons were taken out for cell enumeration analysis.

### Cell Enumeration Analysis

To determine the amount of sessile cells of *V. harveyi* on the AA2024 surfaces, the coupons were placed in a 10 mL centrifuge tube containing 3 mL of sterilized 0.9% NaCl solution, and vortexed for 30 s to detach the sessile cells. Then, 100 μL of the suspension liquid was plated onto a 2216E agar plate (Bartram et al., 2004; Zhao et al., 2016). The plates were incubated at 28°C for 12 h before enumeration. The following equation was used to calculate the antibacterial rate.

$$\begin{array}{c} Antibacteriarate\left(\%\right)=\left(CFU_{c\,o\,n\,t\,r\,o\,l}-CFU_{e\,x\,p\,e\,r\,i\,m\,e\,n\,t\,a\,l\,\,g\,r\,o\,u\,p\,s}\right)/\\ C\,F\,U_{c\,o\,n\,t\,r\,o\,l}\times 100\% \end{array}$$

### Biofilm Observation

After biofilm prevention and removal tests, the biofilms on the coupon surfaces were analyzed using a scanning electron microscope (SEM, Model JSM-6390, JEOL, Tokyo, Japan). The coupons were immersed in 4% (w/w) glutaraldehyde for 4 h to fix the biofilms, and dehydrated using a serial of alcohol solutions (50, 60, 70, 80, 90, 95, and 100% v/v) each for 10 min. Then, the surfaces were sputter-coated with a layer of gold to enhance the surface conductivity before observation.

To analyze the viability of sessile cells in the biofilms, coupons were gently washed with 0.9% NaCl to remove the planktonic cells, stained by a Live/Dead Bacterial Viability Kit (L7012, Life Technologies, Grand Island, NY, United States) for 20 min, and observed using a confocal laser scanning microscope (CLSM, LSM 710, Zeiss, Germany). The live cells were stained by SYTO 9 and shown as green dots at an excitation wavelength of 488 nm. The dead cells were stained by propidium iodide (PI) and shown as red dots at wavelength of 559 nm (Lou et al., 2016).

### ATP Analysis of the *V. harveyi* Biofilm on the Coupon Surfaces

The *V. harveyi* biofilms on the coupon surfaces of AA2024 were wiped using a ATP test paper, and analyzed with a ATP tester (UPF10-ATP, You Pu, China). The luminous value in the same detection range is directly related to the amount of ATP, which determines the quantity of the sessile cells (Wang et al., 2013).

### EPS Staining

The EPSs in the biofilms were quantified using CLSM. The extracellular DNAs, proteins, and polysaccharides in the *V. harveyi* biofilms were stained by 4′,6-diamidino-2-phenylindole (DAPI), Alexa 633 conjugated concanavalin A (ConA-Alexa 633), and SYPRO tangerine, sequentially (Wagner et al., 2009). The excitation and absorption wavelengths for DAPI, ConA-Alexa 633, and SYPRO tangerine were 358/461 nm, 632/647 nm, and 490/640 nm, respectively.

## Results

### *V. harveyi* Biofilm Accelerated the Corrosion of AA2024

**FIGURE 1:**
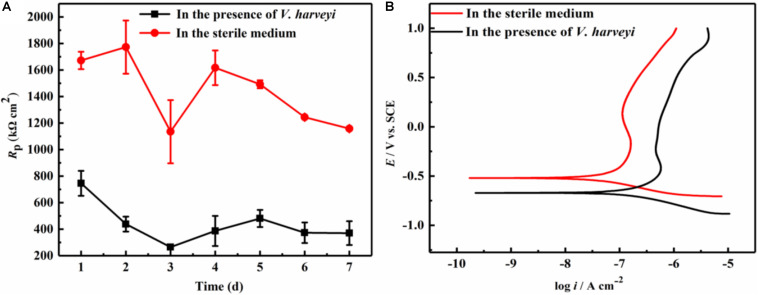
Electrochemical test: **(A)** Variation of *R*_*p*_ values during 7-day immersion, **(B)** Potentiodynamic polarization curves of AA2024 coupons after 7-day immersion.

LPR measurement is a non-destructive electrochemical method, and has been widely used in the study of microbiologically influenced corrosion. Figure 1A demonstrates the variation of polarization resistance (*R*_*p*_) during the 7-day immersion in different culture media. Since the *R*_*p*_ value is inversely proportional to the corrosion rate, the presence of *V. harveyi* significantly decreased the *R*_*p*_ values of AA2024, indicating the accelerated corrosion of AA2024 coupons caused by *V. harveyi* biofilm.

At the end of 7-day immersion in different culture media, potentiodynamic polarization were performed as shown in Figure 1B. The corresponding corrosion parameters were obtained using Tafel fitting analysis and the results are listed in Table 1. The corrosion current density (*i*_*corr*_) in the presence of *V. harveyi* increased significantly to 137.6 ± 11.3 nA cm^–2^, which was approximately 5 times higher than that in the abiotic medium. The corrosion potential (*E*_*corr*_) shifted toward negative direction in the presence of *V. harveyi*, which might result from the oxygen consumption by *V. harveyi* metabolism. The potentiodynamic polarization results further confirmed that corrosion of AA2024 was accelerated by *V. harveyi* biofilm.

**TABLE 1:** Electrochemical parameters obtained from potentiodynamic polarization curves of AA2024 after 7-day immersion.

Medium	*E*_*corr*_ (mV) vs. SCE	*i*_*corr*_ (nA cm^–2^)
In the presence of *V. harveyi*	−610.7 ± 34.9	137.6 ± 11.3
In abiotic medium	−534.5 ± 35.8	28.9 ± 8.4

### Synergistic Effect of D-Amino Acids and CMIT/MIT Prevented the Formation of *V. harveyi* Biofilm

**FIGURE 2:**
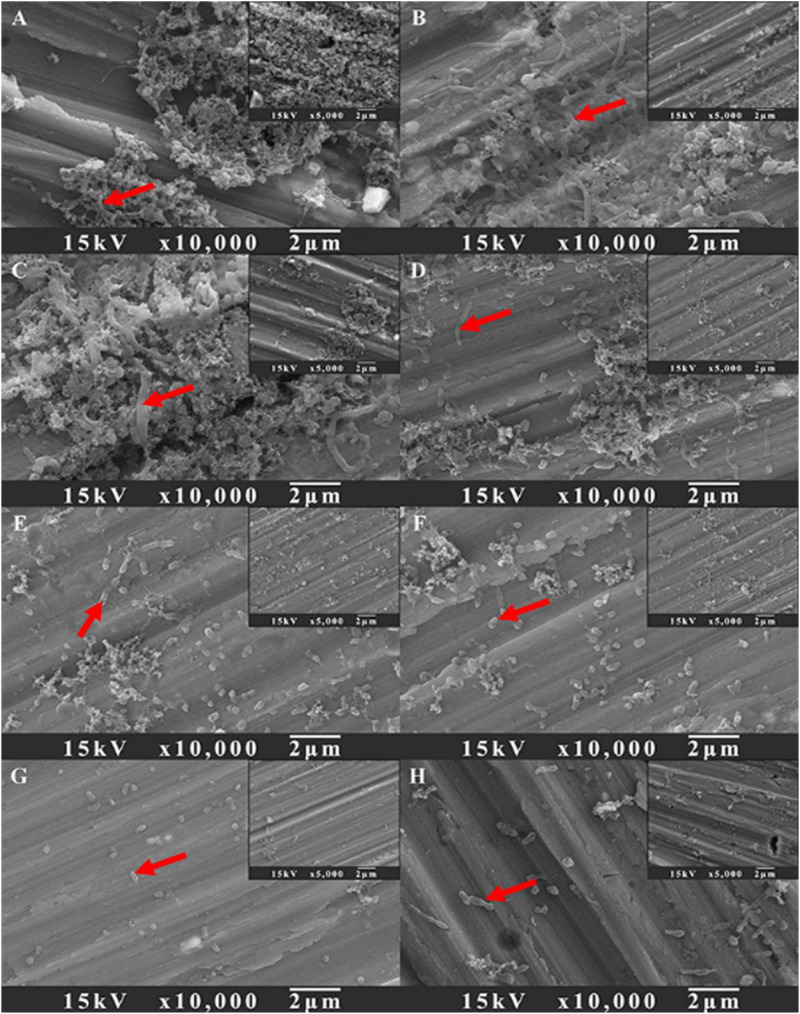
SEM images of sessile cells after 3-day biofilm prevention tests: **(A)** no treatment (control), **(B)** 1 ppm D-Tyr, **(C)** 100 ppm D-Met, **(D)** 5 ppm CMIT/MIT, **(E)** 5 ppm CMIT/MIT + 1 ppm D-Tyr, **(F)** 5 ppm CMIT/MIT + 100 ppm D-Met, **(G)** 5 ppm CMIT/MIT + 1 ppm D-Tyr + 100 ppm D-Met, and **(H)** 25 ppm CMIT/MIT. Red arrows indicate the bacterial cells attached on AA2024 surfaces.

To evaluate the influence of D-amino acids on the prevention of *V. harveyi* biofilm on AA2024 samples, the biofilm prevention test was carried out with different treatment chemicals for 3 days. Figure 2 shows the SEM images of *V. harveyi* sessile cells after treatment with different chemicals for 3 days. Dense biofilms were formed on the surface of AA2024 after incubation for 3 days without any chemical treatment (Figure 2A). Figures 2B,C show that the surfaces of AA2024 were covered with abundant sessile cells, indicating that the treatment of D-amino acids alone did not prevent adhesion and formation of *V. harveyi* biofilm. By contrast, less sessile cells attached on AA2024 coupon after treatment with 5 ppm CMIT/MIT (Figure 2D), demonstrating that the biocide decreased the attachment and formation of *V. harveyi* to a certain extent. Interestingly, only a few sessile cells were detected on the AA2024 coupon surface with dual combination of 5 ppm CMIT/MIT with 1 ppm D-Tyr or 100 ppm D-Met (Figures 2E,F). The treatment with a mixture of 5 ppm CMIT/MIT, 1 ppm D-Tyr and 100 ppm D-Met showed the best efficacy against the attachment and formation of *V. harveyi* biofilm (Figure 2G) which was similar to that obtained with treatment of CMIT/MIT alone at 25 ppm (Figure 2H).

**FIGURE 3:**
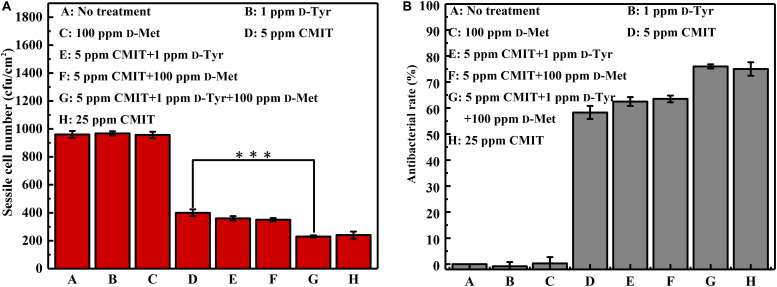
Sessile cell counts after 3-day biofilm prevention test: **(A)** sessile cell number and **(B)** antibacterial rate. Standard deviations were from three independent experiments. ^∗∗∗^*P* < 0.001. There was no significant difference between group G and H (*P* > 0.05).

The data of sessile cell enumeration confirmed the SEM observations (Figure 3). The number of sessile cells on the AA2024 coupon surfaces in the presence of 1 ppm D-Tyr and 100 ppm D-Met alone was not reduced. When treated with 5 ppm CMIT/MIT, the number of sessile cells was decreased over 50%, from 960 to 400 cfu/cm^2^. Moreover, the combination of 1 ppm D-Tyr or 100 ppm D-Met with 5 ppm CMIT/MIT was found to be more effective than the treatment with biocide alone. The best inhibition effect was achieved by the triple combination of 1 ppm D-Tyr, 100 ppm D-Met and 5 ppm CMIT/MIT with a maximum reduction of 76% of the sessile cells. D-amino acids enhanced the antibacterial rate of CMIT/MIT, and with the help of the mixture of 1 ppm D-Tyr and 100 ppm D-Met, the antibacterial rate of 5 ppm CMIT/MIT was basically equivalent to that achieved by 25 ppm CMIT/MIT.

**FIGURE 4:**
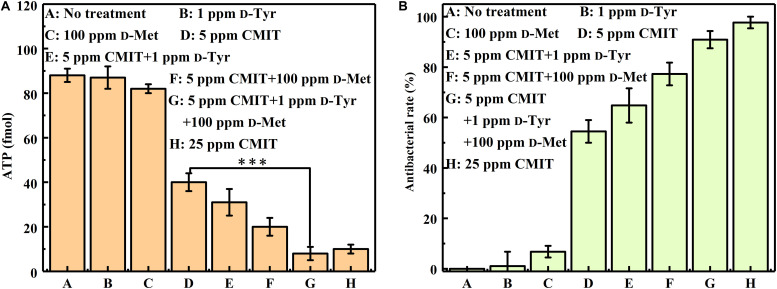
The amount of ATP on the AA2024 coupon surfaces after 3-day biofilm prevention test: **(A)** ATP and **(B)** antibacterial rate. Standard deviations were from three independent experiments. ***P < 0.001. There was no significant difference between group G and H (*P* > 0.05).

As illustrated in Figure 4, the ATP amounts on the AA2024 surface were also obtained to evaluate the efficacy of different treatments. When treated with 5 ppm CMIT/MIT alone, the amount of ATP on the surface of AA2024 was 40 fmol/cm^2^. However, only 8 fmol/cm^2^ ATP was detected on the surface of the AA2024 treated with the triple combination of 1 ppm D-Tyr, 100 ppm D-Met and 5 ppm CMIT/MIT. Again, the prevention effect of the triple combination mixture was similarly identical to that obtained with the concentration of 25 ppm CMIT/MIT (Figure 4).

**FIGURE 5:**
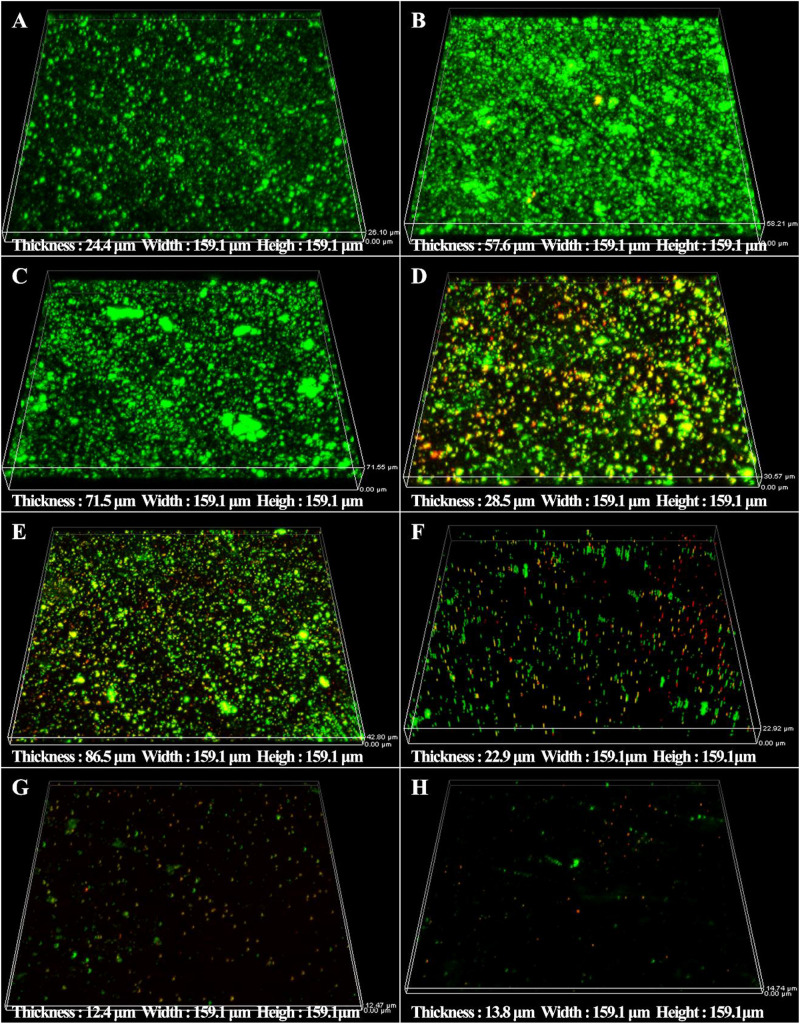
CLSM images of sessile cells after 3-day biofilm prevention test: **(A)** no treatment (control), **(B)** 1 ppm D-Tyr, **(C)** 100 ppm D-Met, **(D)** 5 ppm CMIT/MIT, **(E)** 5 ppm CMIT/MIT + 1 ppm D-Tyr, **(F)** 5 ppm CMIT/MIT + 100 ppm D-Met, **(G)** 5 ppm CMIT/MIT + 1 ppm D-Tyr + 100 ppm D-Met, and **(H)** 25 ppm CMIT/MIT.

The synergistic effect of D-amino acids and CMIT/MIT were also confirmed by Live/Dead staining. Large number of live sessile cells and thick biofilms were observed on the coupon surfaces without treatment (Figure 5A) and treatment with D-Tyr and D-Met alone (Figures 5B,C). Dead cells were observed when 5 ppm CMIT/MIT was treated (Figure 5D). The combination of 100 ppmD-Met with 5 ppm CMIT/MIT achieved a better bactericidal effect compared with the combination of 1ppm D-Tyr and 5 ppm CMIT/MIT (Figures 5E,F). As expected, when the biofilms were treated with 100 ppm D-Met, 1ppm D-Tyr and 5 ppm CMIT/MIT, only a small amount of live cells was observed (Figure 5G), which was almost equivalent to that of using 25 ppm CMIT/MIT (Figure 5H).

**FIGURE 6:**
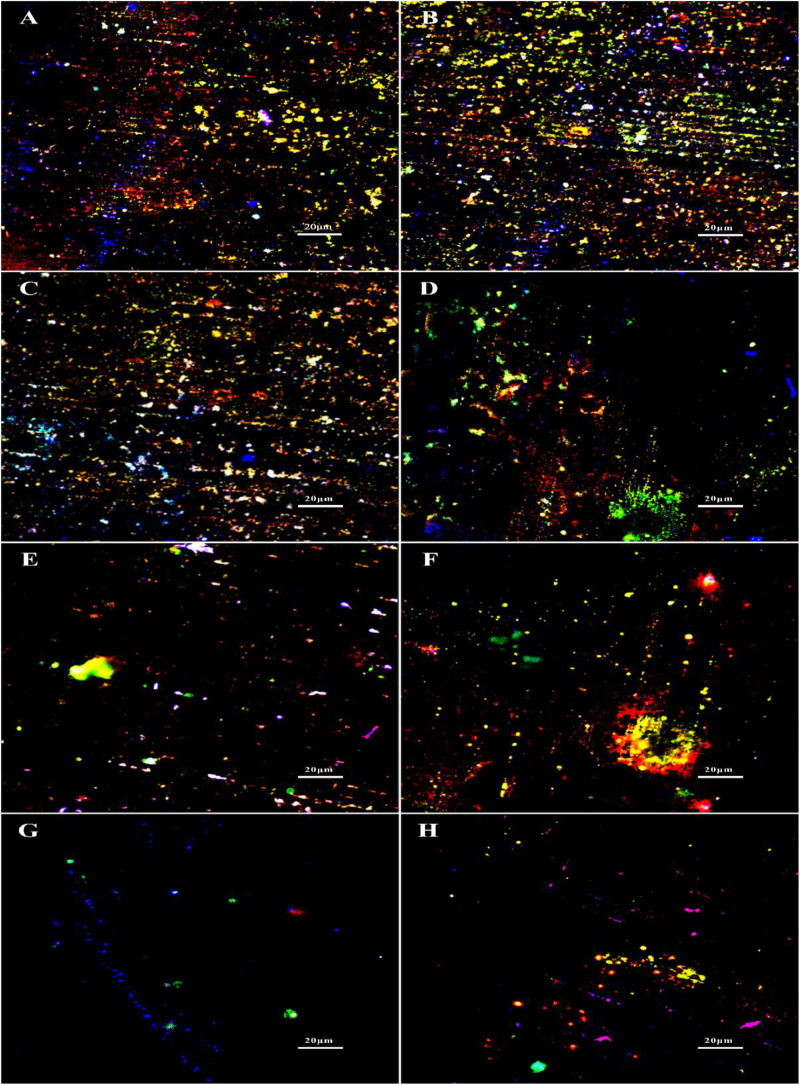
EPS staining after biofilm prevention test: **(A)** no treatment (control), **(B)** 1 ppm D-Tyr, **(C)** 100 ppm D-Met, **(D)** 5 ppm CMIT/MIT, **(E)** 5 ppm CMIT/MIT + 1 ppm D-Tyr, **(F)** 5 ppm CMIT + 100 ppm D-Met, **(G)** 5 ppm CMIT/MIT + 1 ppm D-Tyr + 100 ppm D-Met, and **(H)** 25 ppm CMIT/MIT. Blue, red and green dots represent extracellular DNAs, proteins, and polysaccharides, respectively.

The staining of extracellular DNAs, proteins and polysaccharides (green) in the biofilms treated with different chemicals are shown in Figure 6. Large amounts of DNAs, proteins and polysaccharides were detected in the untreated group, which were similar with the groups treated with 1 ppm D-Tyr and 100 ppm D-Met alone (Figures 6A–C). Whereas, the coupons treated with 5 ppm CMIT/MIT and 1 ppm D-Tyr (or 100 ppm D-Met) showed relatively less amount of EPSs compared to that treated with 5 ppm CMIT/MIT alone (Figures 6D–F). As expected, when the coupons were treated with the mixtures of D-amino acids (100 ppm D-Met and 1 ppm D-Tyr) and 5 ppm biocides CMIT/MIT, only small amount of EPSs were observed on the coupon surfaces (Figure 6G), which was similar to the that of using 25 ppm CMIT/MIT (Figure 6H).

### Synergistic Effect of D-Amino Acids and CMIT/MIT Facilitated the Removal of Mature *V. harveyi* Biofilm

**FIGURE 7:**
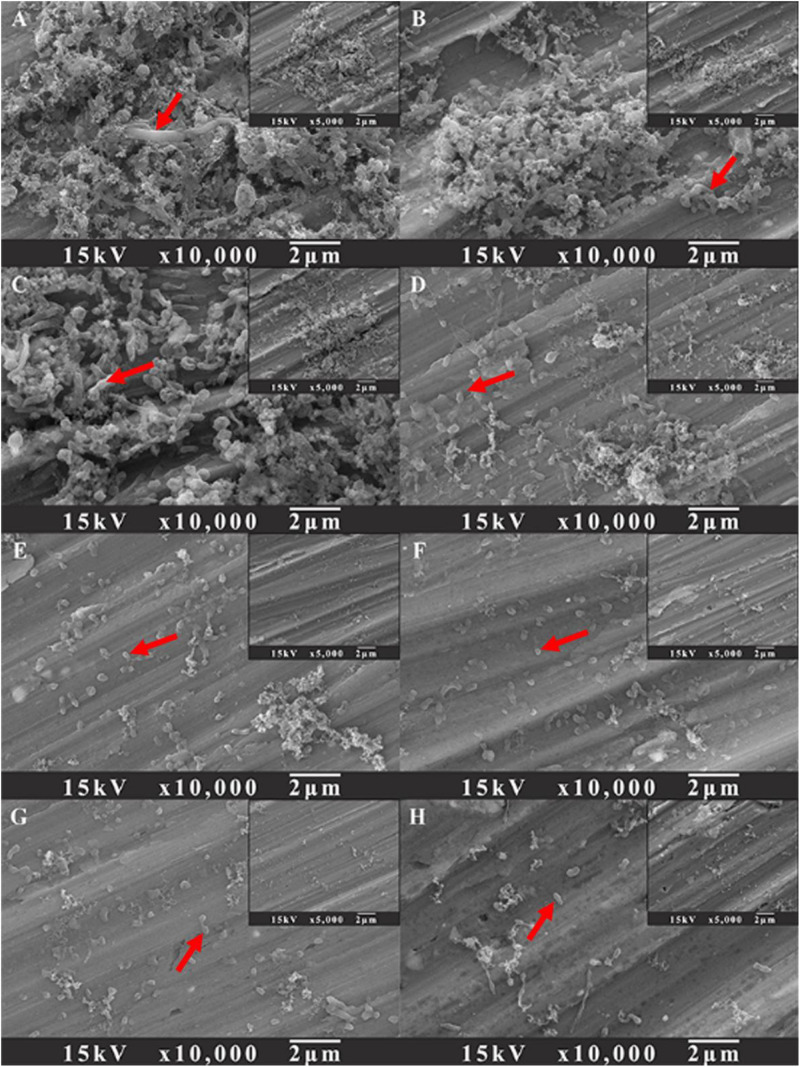
SEM images of sessile cells after the 1-day biofilm removal test: **(A)** no treatment (control), **(B)** 1 ppm D-Tyr, **(C)** 100 ppm D-Met, **(D)** 5 ppm CMIT/MIT, **(E)** 5 ppm CMIT/MIT + 1 ppm D-Tyr, **(F)** 5 ppm CMIT + 100 ppm D-Met, **(G)** 5 ppm CMIT/MIT + 1 ppm D-Tyr + 100 ppm D-Met, and **(H)** 25 ppm CMIT/MIT. Red arrows indicate the bacterial cells attached on AA2024 surfaces.

SEM images of sessile cells on AA2024 coupon surfaces after 1-day biofilm removal treatment are shown in Figure 7. For the untreated coupons and coupons treated with 100 ppm D-Met or 1 ppm D-Tyr alone, a large quantity of sessile cells were detected (Figures 7A–C). However, when the coupons were treated by combination of 5 ppm CMIT/MIT with 100 ppm D-Met (Figure 7F) or 1 ppm D-Tyr (Figure 7E), the amount of sessile cells was reduced significantly, compared to that treated by 5 ppm CMIT/MIT alone (Figure 7D). When the sessile cells were treated with the triple combination of 5 ppm CMIT/MIT, 100 ppm D-Met, and 1 ppm D-Tyr (Figure 7G), few sessile cells were observed on the coupon surface, achieving the removal efficacy equivalent to the treatment with 25 ppm CMIT/MIT (Figure 7H).

**FIGURE 8:**
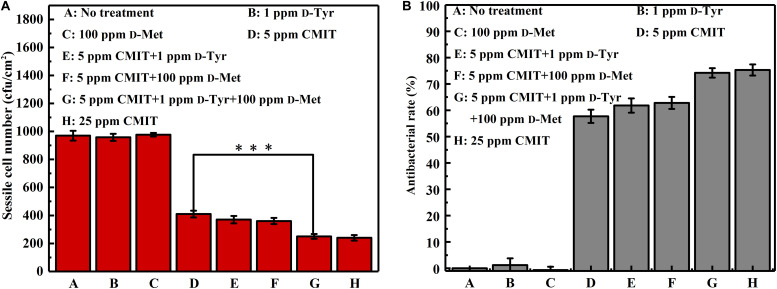
Sessile cell counts on the AA2024 coupon surfaces after 1-day biofilm removal test: **(A)** sessile cell number and **(B)** antibacterial rate. Standard deviations were from three independent experiments. ***P < 0.001. There was no significant difference between group G and H (*P* > 0.05).

**FIGURE 9:**
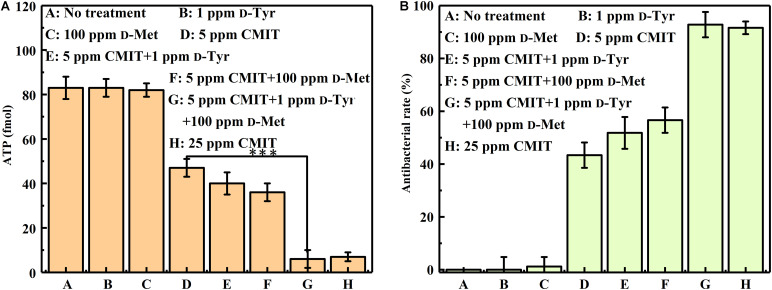
The amount of ATP on the AA2024 coupon surfaces after 1-day biofilm removal test: **(A)** ATP and **(B)** antibacterial rate. Standard deviations were from three independent experiments. ***P < 0.001. There was no significant difference in group G and H (*P* > 0.05).

Figure 8 shows a synergistic effect between D-amino acids and CMIT/MIT on the mitigation of *V. harveyi* biofilms. Without chemical treatment, 970 cfu/cm^2^ sessile cells on the coupon surface were observed, and 100 ppm D-Met and 1 ppm D-Tyr alone could hardly remove the sessile cells from the coupon surface (Figures 8, groups A to C). When 5 ppm CMIT/MIT was applied, 410 cfu/cm^2^ sessile cells were detected on the coupon surface, indicating that more than half of the sessile cells were removed. With the combination of D-amino acid (whether 100 ppm D-Met or 1 ppm D-Tyr) and 5-ppm CMIT/MIT, less sessile cells were detected on the coupon surfaces. With the triple combination of 100 ppmD-Met, 1 ppm D-Tyr and 5 ppm CMIT/MIT, the sessile cells on the coupon surface decreased to 250 cfu/cm^2^, which was almost the same as that achieved using 25 ppm CMIT/MIT. Furthermore, the ATP amounts on the coupon surfaces were also determined to reflect the quantity of the biofilm on the coupon surfaces after different treatments (Figure 9). As expected, the lowest ATP level (6 fmol/cm^2^) was detected for the treatment with 100 ppmD-Met, 1 ppm D-Tyr and 5 ppm CMIT/MIT (Figure 9, group G).

**FIGURE 10:**
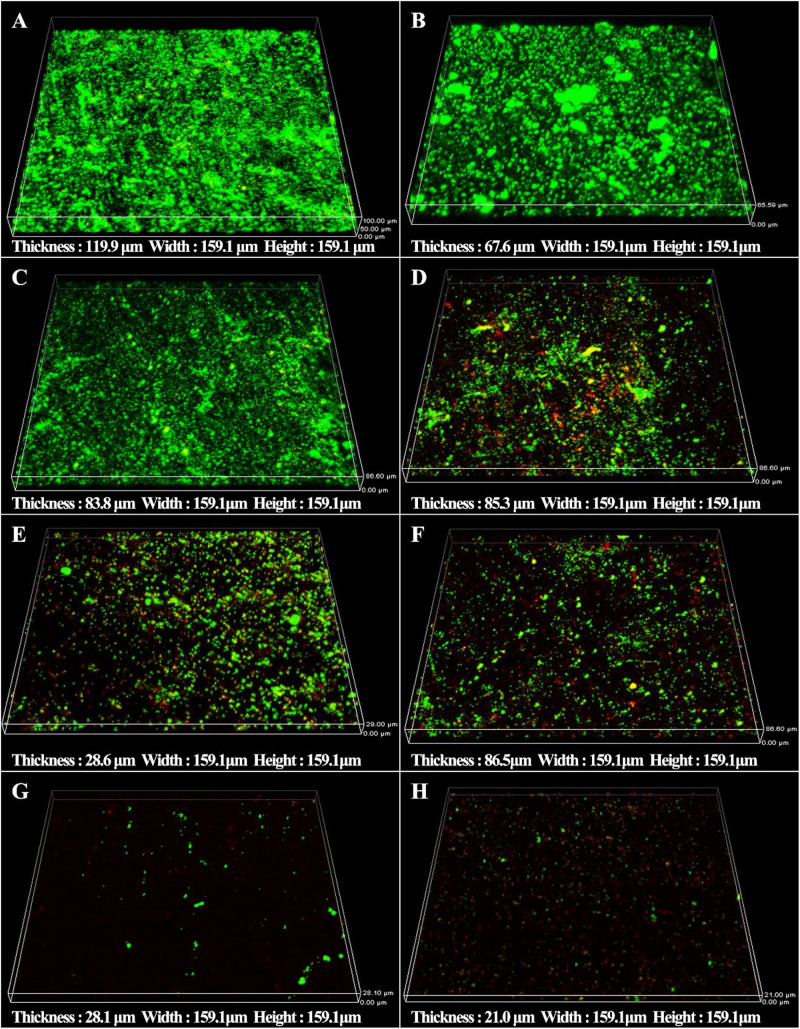
CLSM images of sessile cells after 1-day biofilm removal test: **(A)** no treatment (control), **(B)** 1 ppm D-Tyr, **(C)** 100 ppm D-Met, **(D)** 5 ppm CMIT/MIT, **(E)** 5 ppm CMIT/MIT + 1 ppm D-Tyr, **(F)** 5 ppm CMIT/MIT + 100 ppm D-Met, **(G)** 5 ppm CMIT/MIT + 1 ppm D-Tyr + 100 ppm D-Met, and **(H)** 25 ppm CMIT/MIT.

The sessile cell counts were supported by the Live/Dead staining results as demonstrated in Figure 10. For the untreated coupons and coupons treated with 100 ppm D-Met or 1 ppm D-Tyr alone, a large number of live sessile cells was observed (Figures 10A–C). Much more sessile cells on AA2024 coupon surfaces were killed when treated with the combination of 5 ppm CMIT/MIT and 1 ppm D-Tyr (or 100 ppm D-Met) compared with biocide treatment alone (Figures 10E,F). When 5 ppm CMIT/MIT, 100 ppm D-Met and 1 ppm D-Tyr were used together, the least live sessile cells were observed, which was similar to that of using 25 ppm CMIT/MIT (Figures 10G,H).

**FIGURE 11:**
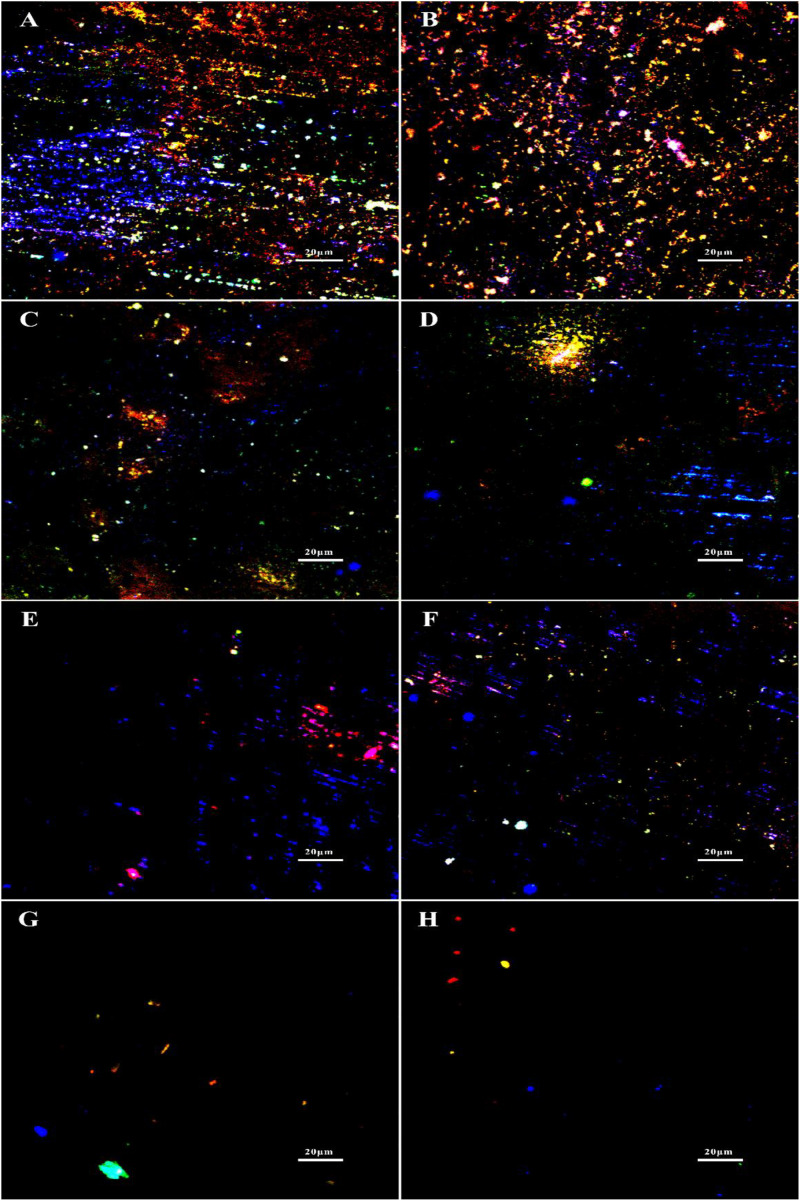
EPS staining after 1-day biofilm removal test: **(A)** no treatment (control), **(B)** 1 ppm D-Tyr, **(C)** 100 ppm D-Met, **(D)** 5 ppm CMIT/MIT, **(E)** 5 ppm CMIT/MIT + 1 ppm D-Tyr, **(F)** 5 ppm CMIT/MIT + 100 ppm D-Met, **(G)** 5 ppm CMIT/MIT + 1 ppm D-Tyr + 100 ppm D-Met, and **(H)** 25 ppm CMIT/MIT. Blue, red, and green dots represent extracellular DNAs, proteins, and polysaccharides, respectively.

The synergistic effect of D-amino acids and CMIT/MIT on the removal of mature *V. harveyi* bifilm was further confirmed by EPS staining (Figure 11). It can be seen that the untreated coupons and coupons treated with 100 ppm D-Met (or 1 ppm D-Tyr) were abundant with sessile cells and EPSs (Figures 11A–C). When 5 ppm biocide CMIT/MIT was used, and the amount of EPSs decreased significantly. After treatment with the combination of 5 ppm CMIT/MIT and 1 ppm D-Tyr (Figure 11E) or 100 ppmD-Met (Figure 11F), the amount of EPSs on the coupon surfaces was much less than that treated with 5 ppm CMIT/MIT alone (Figure 11D). When treating with the combination of 5 ppm CMIT/MIT, 100 ppmD-Met, and 1 ppm D-Tyr (Figure 11G), the most efficient removal of *V. harveyi* biofilm was achieved, which was similar to that of using 25 ppm CMIT/MIT (Figure 11H).

## Discussion

*V. harveyi* is commonly found in the aircraft fuel tank, causing microbial contamination. According to the obtained electrochemical data, we first confirmed the accelerated corrosion caused by *V. harveyi*.

It is well known that the biofilms attached on the metal surfaces rather than the planktonic counterparts are responsible for microbiologically influenced corrosion (MIC) (Xu et al., 2016). The metabolic activity of the sessile cells embedded in biofilms causes the change of electrochemical parameters (such as pH, oxygen, and ionic strength) underneath the biofilms, resulting in the accelerated corrosion of metal materials. Moreover, recent progress in MIC confirmed that the extracellular electron transfer (EET) between the metal matrix and the biofilm played the dominant role in the corrosion process caused by electroactive biofilms (Jia et al., 2019; Dong et al., 2020). In this study, electrochemical tests were performed to evaluate the corrosion behavior of AA 2024 in the presence of *V. harveyi*. The decrease of polarization resistance (Figure 1A) and increase of corrosion current density (Table 1) demonstrated that *V. harveyi* biofilm accelerated the corrosion of AA 2024. Further work is desired to investigate mechanism of MIC caused by *V. harveyi*.

Microbial contamination of the aircraft fuel system has been long recognized and considered as a thorny problem in assuring the service safety of aircraft (Raikos et al., 2011). Repeated treatment with biocides is frequently resorted to control and mitigate the attached biofilms. However, more efficient methods are still desirable to reduce the dosage and overuse of biocides. The triple combination of 1 ppm D-Tyr and 100 ppmD-Met significantly increased the efficacy of 5 ppm CMIT/MIT in both prevention and removal of *V. harveyi* biofilms, considerably reducing the biocide dosage.

**FIGURE 12:**
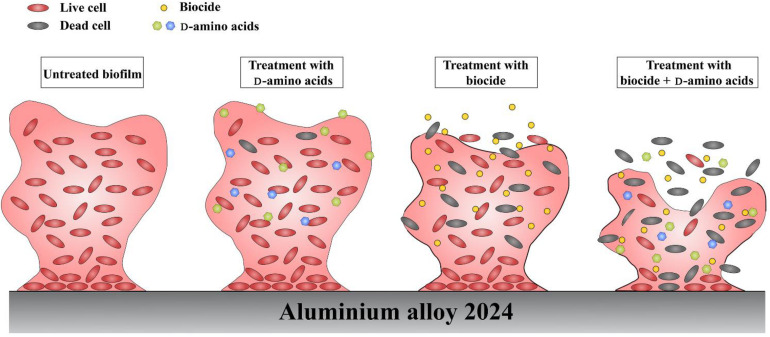
Schematic illustration of the synergy between D-amino acids and biocide.

Currently, the dispersal mechanisms of D-amino acids have not been fully understood, however, several possible mechanisms have been proposed. Kolodkin-Gal et al. (2010) believed that the D-alanine terminus in peptidoglycans might be replaced with D-amino acids, causing structural change which prevented the binding of protein onto the cell wall and caused the disassembly of bacterial biofilms. Since peptidoglycans are the basic components of most bacterial cell walls, D-amino acids are effective toward a wide-spectrum of bacterial biofilms. D-amino acids are able to disperse and disassemble the biofilm, but they are not biocidal, which was verified in this work and previous report (Xu et al., 2014). It is well known that biofilms are more tenacious to treat than its planktonic counterparts. Thus, D-amino acids can act as a biofilm disassembly signal, converting the sessile cells into planktonic cells which are easier to be killed in the bulk fluid. With the help of biocides, D-amino acids can work as excellent biocide enhancers. In the study, it is found that the mixture of D-Tyr and D-Met can convent the difficult-to-kill biofilms into easy-to-eradicate planktonic cells, so that D-amino acids enhance the biocidal efficacy of CMIT/MIT against *V. harveyi*. In view of this, we proposed the schematic illustration for the synergistic effect between D-amino acids and CMIT/MIT (Figure 12). The synergistic effect between D-amino acids and biocide improved the efficacy of antimicrobial agents, resulting in a similar biocidal efficacy identical to a much higher biocide concentration.

Previously, Ramon-Perez et al. (2014) demonstrated the remarkable differences of different *Staphylococcus epidermidis* strains in sensitivities and resistances toward different D-amino acids. It has been shown that D-Tyr, D-Leu, D-Try and D-Met were able to inhibit the formation of *Bacillus subtilis* and *Pseudomonas aeruginosa* biofilms (Kolodkin-Gal et al., 2010). In contrast, the formation of *Staphylococcus aureus* SC01 biofilm was not affected by those D-amino acids, but hampered by D-Phe, D-Pro, and D-Tyr (Hochbaum et al., 2011). These results suggested that the compositions of biofilms varied among different bacteria. Consequently, it is important to use D-amino acids mixtures to achieve excellent efficacy. Our results here show that the formation prevention and removal of *V. harveyi* biofilms were both impeded by a mixture of D-Met and D-Tyr. Meanwhile the combination of D-Met and D-Tyr further improved the biocidal efficacy of CMIT/MIT against *V. harveyi* biofilm. It is possible that the *V. harveyi* biofilm is also susceptible toward other untested D-amino acids or their mixtures.

## Conclusion

We first reported that the *V. harvey*i biofilm can cause MIC of AA2024. D-Met and D-Tyr can increase the biocidal effect of CMIT/MIT against the *V. harveyi* biofilm individually, while a mixture of 100 ppm D-Met and 1 ppm D-Tyr significantly enhanced the antibacterial efficacy of 5 ppm CMIT/MIT, resulting in an identical effect to that obtained with the concentration of 25 ppm CMIT/MIT. D-amino acids possess potent biofilm disassembly properties as a green and efficient biocide enhancer in aviation fuel system. Optimization of more D-amino acids mixtures with better biofilm dispersal ability and broader spectrum is desired.

## Data Availability Statement

The raw data supporting the conclusions of this article will be made available by the authors, without undue reservation.

## Author Contributions

XL conducted the experiments, analyzed the data, and drafted the manuscript. ZL analyzed the data and reviewed the manuscript. YS and DX conceived the study. DX guided the research and polished the manuscript critically. YF, YL, LD, and ZZ assisted the experiment design. FW directed the experiment. All the authors approved the final manuscript.

## Conflict of Interest

The authors declare that the research was conducted in the absence of any commercial or financial relationships that could be construed as a potential conflict of interest.
